# Alveolar Abscess

**Published:** 1869-06

**Authors:** 


Alveolar Abscess.—Prof. Judd (*Missouri Dental Journ.*) treats incipient alveolar abscess successfully by long continued freezing of the parts with Richardson's apparatus. The tooth should be frozen its whole length, which will take from fifteen to thirty minutes. Immediate relief from the pain will ensue, and in many cases it will not return.

